# Tengdan Capsule Prevents Hypertensive Kidney Damage in SHR by Inhibiting Periostin-Mediated Renal Fibrosis

**DOI:** 10.3389/fphar.2021.638298

**Published:** 2021-05-18

**Authors:** Xiaoli Du, Qianqian Tao, Hongxia Du, Zhenbang Zhao, Yu Dong, Shuang He, Rui Shao, Yule Wang, Wenrun Han, Xintong Wang, Yan Zhu

**Affiliations:** ^1^ Institute of Chinese Medicine, Tianjin University of Traditional Chinese Medicine, Tianjin, China; ^2^ Department of pharmacy, Inner Mongolia Medical College, Hohhot, China; ^3^ Tianjin International Joint Academy of Biomedicine, Tianjin, China

**Keywords:** hypertension-induced kidney damage, periostin, TGFβ/SMAD signaling pathway, inflammatory response, human kidney HEK293 cells

## Abstract

**BACKGROUND:** Hypertension-induced renal damage is a serious and complex condition that has not been effectively treated by conventional blood pressure-lowering drugs. Tengdan capsule (TDC) is a China FDA-approved compound herbal medicine for treating hypertension; however, its chemical basis and pharmacological efficacy have not been fully investigated in a preclinical setting.

**METHODS:** High-performance liquid chromatography (HPLC) was used to identify and quantify the major chemical components of TDC extracted from ultrapure water. Adult spontaneously hypertensive rats (SHR) and age/sex-matched Wistar Kyoto normotensive rats (WKY) were both treated with TDC, losartan, or saline for one month, and their blood pressure (BP) was monitored at the same time by tail-cuff BP system. Biochemical indexes such as urine creatinine (CRE) and blood urea nitrogen (BUN) were determined. Kidney tissue sections were examined with (H&E), and Masson staining to evaluate the pathological effect of TDC on SHR’s kidneys. After TDC treatment, the differentially expressed proteins in the kidneys of SHR were identified by the TMT-based quantitative proteomics analysis, which may provide the targets and possible mechanisms of TDC action. In addition, Western blot analysis, RT-qPCR, and ELISA assays were carried out to further verify the proteomics findings. Finally, two different models involving *in vitro* renal injuries were established using human kidney HEK293 cells; and the molecular mechanism of TDC kidney protection was demonstrated.

**RESULTS:** Seven chemical compounds, namely Notoginsenoside R1, Ginsenoside RG1, Ginsenoside Re, Ginsenoside Rb1, Sodium Danshensu, Protocatechualdehyde, and Salvianolic acid B, were identified and quantified from the water-soluble extracts of TDC by HPLC. *In vivo* study using rats showed that TDC effectively reduced BP, BUN, and CRE levels and attenuated renal fibrosis in SHR, and ameliorated damage to the kidneys. Proteomics and subsequent bioinformatics analyses indicated that periostin-mediated inflammatory response and TGFβ/Smad signaling pathway proteins were closely related to the therapeutic effect of TDC in rat kidneys. Western blot analysis and RT-qPCR showed that TDC markedly downregulated the mRNA and protein expression of periostin in renal tissues compared to the untreated SHR. In addition, TGF-β and COL1A1 mRNA levels also decreased in SHR renal tissues following TDC treatment. *In vitro* studies showed that low to medium doses of TDC down-regulated the expression of periostin in the injury model of HEK293 cell. In addition, medium to high doses of TDC significantly inhibited collagen deposition in TGFβ1-induced HEK293 cell fibrosis.

**CONCLUSIONS:** Major components from the compound herbal medicine Tengdan Capsule are identified and quantified. TDC effectively lowers blood pressure and protects against renal damage caused by hypertension in SHR. Mechanistically, TDC blocks periostin by regulating the TGF-β/Smad signaling pathway in the kidney, both *in vivo* and *in vitro*. Preventing periostin-mediated renal fibrosis and inflammation might be a promising strategy for treating a hypertensive renal injury.

## Introduction

Hypertension is a common disease and a major risk factor for multiple organ abnormalities (Lackland and Weber, 2015; Poulter et al., 2015). The kidney is an important organ for excreting wastes and maintaining the water balance, electrolytes, acid, and base. It plays a role in maintaining normal BP and in the pathogenesis of hypertension. Hypertension is also an important risk factor for the progressive decline of renal function in patients with kidney disease (Griffin, 1979).

Hypertension causes renal damage mainly through two pathways, one is ischemic changes of renal tissue caused by renal artery stenosis caused by arterial hypertension, and the other is renal tissue damage caused by renal hemodynamic abnormalities. Progression through inflammation and fibrosis eventually leads to mesangial matrix hyperplasia, renal tubular atrophy, interstitial fibrosis, and glomerulosclerosis (Mennuni et al., 2014). Renal fibrosis is a common contributor to the progression of various chronic kidney diseases to end-stage renal failure (Liu and Zhuang, 2019). Manotham et al. found that renal tubular epithelial cells and stromal fibroblasts could transform into myofibroblasts phenotypes and participate in the development of renal interstitial fibrosis (Manotham et al., 2004). Myofibroblasts have an active ability to proliferate and secrete collagen and are the main source of increased deposition of extracellular matrix (ECM). Epithelial-to-mesenchymal transition (EMT) plays an important role in the pathogenesis of renal interstitial fibrosis (Farris and Colvin, 2012) and α-SMA is now considered a marker of myofibroblast, and its expression can be used to predict the degree of fibrosis (Rodemann and Müller, 1990; Mack and Yanagita, 2015). Transforming growth factor-β (TGF-β) is a key driver of renal fibrosis, especially when activating renin-Ang system (RAS), which is the main cause of hypertension (Kohan et al., 1989; Kagami et al., 1994). TGF-β induces renal fibrosis by increasing the deposition of extracellular matrix proteins and inhibiting the activity of matrix metalloproteinases (Border, 1994; Douthwaite et al., 1999; Mozes et al., 1999). In the past few years, several studies have identified periostin as a new key player in the progression of kidney disease. Animal model studies have shown that periostin plays a central role in mediating renal inflammation and fibrosis, leading to the deterioration of renal structure and function. Periostin expression levels in kidneys and urine are also highly correlated with pathological stage and renal function decline in patients with renal disease in different age groups (Prakoura and Chatziantoniou, 2017).

Tengdan Capsule (TDC, China Food and Drug Administration National drug approval number Z20133012), produced by Shanxi Buchang HI-TECH Pharmaceutical Co., LTD., is a pure herbal compound made into a capsule, composed of the following ten flavor of traditional Chinese medicine: *Rubiaceae; Uncaria rhynchophylla* (17.10%), *Lamiaceae; Prunella vulgaris* (11.90%), *Pork bile Electuary* (1.82%), *Loranthaceae; Scurrula parasitica* (14.52%), *Lamiaceae; Radix Salviae miltiorrhizae* (12.02%), *Conioselinum anthriscoides* ‘*Chuanxiong*’ (7.94%), *Panax pseudoginseng Wall* (1.82%), *Plantago asiatica L* (9.08%), *Stephania tetrandra S. Moore* (11.90%) and *Astragalus mongholicus Bunge* (11.90%) (Zhao, 2005). Tengdan Capsule is prescribed in hospitals and pharmacies in China for treating mild and moderate hypertension, with TCM syndrome indications including promoting blood circulation to remove blood stasis and clearing damp to remove phlegm (Shandong Buchang Pharmaceutical Co., Ltd. Manufacture’s guide of Tengdan capsule, 2001). Although the preclinical research is scanty, the blood pressure lowing effect of TDC in SHR was reported (Hu et al., 2002). Antihypertensive therapy research on Chinese herbal formulas for treating hypertension has made rapid progress over the past 40 years (Xiong et al., 2013), but the mechanistic insights of multi-component compound formulas multi-targeting a complex disease are still very limited. We have shown previously that a two-component compound formula composed of *Radix Salvia miltiorrhizae* and *Carthamus tinctorius*, Danhong injection, reduces vascular remodeling and up-regulates the kallikrein-kinin system in spontaneously hypertensive rats (Yang et al., 2017). Importantly, Danhong injection protects hypertension-induced renal injury via downregulation of myoglobin expression in spontaneously hypertensive rats (Owoicho Orgah et al., 2018).

The present study aimed to define the major chemical ingredients and to investigate the pharmacodynamic effect of TDC on renal injury in hypertensive rats. Based on the quantitative proteomic analysis of Tandem Mass Tag (TMT), candidate target proteins of TDC in hypertensive kidney were identified, and both *in vivo* and *in vitro* experiments were conducted to reveal that TDC reduces renal damage (fibrosis) in hypertensive rats by inhibiting the periostin-mediated inflammatory response.

## Materials and Methods

### Drugs and Reagents

Tengdan Capsule (TDC) (Drug approval number: Z20133012; Batch number:231101101) was supplied by Shanxi Buchang HI-TECH Pharmaceutical Co., LTD. The standard Notoginsenoside R1 (S-002-180807), Ginsenoside RG1 (R-015-181016), Ginsenoside Re (R-020-180426), Ginsenoside Rb1 (R-012-180314), Sodium Danshensu (110855-200508), Protocatechualdehyde (110810-200506) and Salvianolic acid B (111562-200605) was purchased from China Institute for Drug and Biological Products Inspection. Losartan potassium tablets (LST) (drug approval number: H20080371, Batch No.19022806) was purchased from Yangzijiang Pharmaceutical Group (Sichuan, China). Acetonitrile, methanol (HPLC grade), and phosphoric acid were supplied by Fisher Scientific (Shanghai, China). Other reagents and deionized water were the analytical grades. Assay kits for creatinine (CRE) and blood urea nitrogen (BUN) were purchased from Jiancheng Bioengineering Institute (Nanjing, China).RIPA tissue/cell lysis buffer (R0020), BCA (PC0020) Protein Assay Kit, SDS-PAGE Gel Kit (20190919), Albumin Bovine V (80810511), and Paraformaldehyde (4%) (P1110-500 ml) were purchased from Solarbio Science &Technology Co., Ltd. (Beijing, China). Rabbit anti-periostin polyclonal antibody was purchased from Bioss (bs-4994R, Beijing, China). Anti-alpha smooth muscle actin antibody [1A4] ab7817 was purchased from Abcam (Shanghai, China). Sirius Red staining kit (200405) was purchased from Nanjing SenBeiJia Biological Technology Co., Ltd. Isoflurane (Lot No. B506) was obtained from Ruiwode Lifescience Co., Ltd (Shenzhen, China). Goat anti-rabbit IgG H&L antibody was obtained from Zhongshan Jingqiao Biotechnology, Inc. (ZB-5301, Beijing, China). POSTN ELISA kit was purchased from ZCi Bio Co., Ltd (Shanghai, China). Sodium dodecyl sulfate (SDS) (161-0302), DL-dithiothreitol (DTT) (161-0404), Iodoacetamide (IAA) (163-2109), and urea (161-0731) were obtained from Bio-Rad (Hercules, CA). Tris (A6141), C18 Cartridge (66872-U), and Trifluoroacetic Acid (TFA) (T6508) were obtained from Sigma (St. Louis, MO, United States). SDT (4%SDS, 100 mm Tris-HCl, 1 mm DTT, pH7.6) lysis Buffer, 5X Loading Buffer (10% SDS,0.5% Bromophenol Blue,50% Glycerol, 500 mm DTT, 250 mm Tris-HCl, pH6.8). Recombinant Human TGF-β1 protein was purchased from the R&D system (240-B-002, Beijing, China). Sirius Red staining kit was purchased from SenBeiJia BioTech Co., Ltd. (SBJ-0294, Nanjing, China).

### Experimental Animals

Age and weight (240 ± 10 g) matched male 12-week-old spontaneously hypertensive rats (SHR) and male Wistar-Kyoto rats (WKY) were purchased from Beijing Vital River Laboratory Animal Technology Co., Ltd. (Beijing, China, Certificate No. SCXK Jing 2017-0005) and used as experimental hypertensive rat model and the normotensive rat control respectively. All experimental animals were treated in accordance with the guidelines of The Experimental Animal Ethics Committee of Tianjin University of Traditional Chinese Medicine (License No. TCM-LAEC2016034) and Tianjin International Joint Academy of Biomedicine (License No. TJU20160024). Rats were housed under room temperature (22 ± 2°C) and relative humidity (40 ± 5%) conditions with 12 h dark/light cycles. Apart from WKY group, the selected experimental SHR were randomly divided into five different groups, including SHR (model), LST (losartan positive control, 10 mg/kg), TDC low dose (0.6 g/kg) +SHR, middle dose (1.2 g/kg) +SHR, and high dose (2.4 g/kg) +SHR groups. Our pilot study showed that the anti-hypertensive effect of medium dose (1.2 g/kg) was more prominent and the rats with this concentration had a high acceptance degree by gavage administration (data are not shown). Therefore, the medium dose was chosen for the rest of the *in vivo* study. TDC (1.2 g/kg) and LST (10 mg/kg) were dissolved in pure water with ultrasound, and the rats were given intragastric administration, once a day, for consecutive 28 days. Meanwhile, the WKY and SHR model groups were given 0.9% normal saline *via* gavage at a volume of 10 ml/kg. Blood pressure was monitored weekly. CRE and BUN levels were determined in the urine after 4 weeks. Before the surgery, rats were anesthetized with 4% isoflurane in 70% nitrous oxide (N _2_O)/30%oxygen (O_2_). Then, isoflurane was lowered to 2.5% to maintain anesthesia, using a small animal anesthesia machine (Matrix VIP 3000; Midmark, United States). Kidney tissues were harvested, with one part placed in 10% formalin for H&E staining and immunohistochemical analysis, and the other part frozen quickly in liquid nitrogen for proteomics, qRT-PCR validation, and Western Blot analysis.

### HPLC Analysis of Water-Soluble Extracts of TDC

#### Sample Preparation

Mixed standards (MS) were prepared by dissolving an appropriate amount of the compounds in methanol and filtered by 0.20 membrane. MS1 contained Danshensu sodium, protocatechuic aldehyde, and Salvianolic acid B, MS2 contained Notoginsenoside R1, Ginsenoside RG1, Ginsenoside Re, Ginsenoside Rb1. For sample solutions, an appropriate amount of Tengdan capsule was taken and finely ground. After passing an 80 target quasi-sieve, 0.6 g powder was accurately weighed and dissolved in 20 ml distilled water to form 30 mg/ml, and sealed for 30 min with ultrasonic (frequency: 40 K Hz, power: 500 w, temperature: 60°C), and filtered through 0.20 μm membranes.

### Chromatographic Conditions

Determination of water-soluble active components in TDC was carried out using a Thermo U-3000 series HPLC system equipped with an Lpg-3400sd pump, WPS-3000 automatic sampler, TCC-3100 column, and photodiode array detector (or diode array detector). Chromatographic separation was achieved on an Elit reversed-phase column C 18 column (4.6 × 150 mm, 5 μm) maintained at 30°C. Methanol (solvent A) and 0.1% phosphoric acid in water (solvent B) served as one of the mobile phases. The gradient program is as follows: 0–10 min, 5–10% A; 10–20 min, 10–20% A; 20–30 min, 20–30% A; 30–40 min, 30–40% A; 40–50 min, 40–50% A; 50–55 min, 55–5% A; 55–65 min, remain 5% A. The detection wavelength was set at 245 nm. Acetonitrile (solvent A) and deionized water (solvent B) served as another mobile phase. The gradient program was as follows: 0–22 min, 19% A; 22–60 min, 19–36% A; 60–65 min, 36–19% A; 65–75 min, remain19% A. The detection wavelength was set at 203 nm. A flow rate of 1.0 ml/min was utilized. An aliquot of 20 μl sample solution was injected for analysis.

### Blood Pressure Measurement

As previously reported (Yang et al., 2017), 8-channel CODA non-invasive blood pressure (BP) collection system (Kent Scientific Corporation, CT, United States) was used to measure the tail arterial pressure before and after the drug administration. Rats were trained for a week on the device prior to the measurement. The sphygmomanometer was preheated for 5–10 min at a constant temperature of 40°C before the rats were put into the heat preservation sleeve of the blood pressure monitor. The value of the caudal artery BP was measured using a pressure sensor, inserted from the tip of the tail, and advanced towards the base of the tail. Systolic, diastolic, mean BP, and heart rate were recorded. In the awake and quiet state, the rats were continuously measured four times and the average value was taken.

### Estimation of CRE and BUN Levels

Creatinine (CRE) and Blood urea nitrogen (BUN) contents were determined using commercial kits according to the manufacturer’s instruction (Jiancheng Biomedicine, China). CRE and BUN concentrations were also determined according to the instruction.

### Hematoxylin and Eosin Staining

Kidney specimens of each animal were fixed with 10% formalin for more than 48 h, paraffin-embedded, sliced with a manual microtome (HM355S, Thermo., Ltd., United States) with a thickness of 4 µm (Jing et al., 2011), and then put into an automatic staining machine (Gemini, Thermo., Ltd., United States) for HE staining. Pathological changes of renal tissue were observed by optical microscope (DP71, OLYMPUS, Japan).

### Masson Staining

The tissue sections were stained with Masson trichromatic staining kit (Solarbio, China, Catalog No. G1340) to detect renal collagen deposition. Morphological and pathological changes of the kidney were recorded under an optical microscope (DP71, OLYMPUS, Japan).

### TMT-Based Quantitative Proteomics Analysis

#### Sample Preparation

Rats from the model group and TDC (0.24 g/ml) group (*n* = 3 each) were chosen for proteomic analysis. After 4 weeks of TDC administration, the rats were anesthetized and the kidney tissue was immediately removed, then snap-frozen in liquid nitrogen and grounded with a pestle and mortar. Five times volume of TCA (Trichloroacetic acid)/acetone (1:9) was added to the powder and mixed by a vortex. The mixture was placed at -20°C for 4 h, and centrifuged at 6000 g for 40 min at 4°C. The supernatant was discarded. The pre-cooling acetone was added to the pellet and washed three times. The precipitation was air-dried. A 30-times volume of SDT buffer was added to 20–30 mg powder, mixed, and boiled for 5 min. The lysate was sonicated and then boiled for 15 min. After centrifugation at 14,000 *g* for 40 min, the supernatant was filtered with 0.22 µm filters. Total protein in the filtrate was quantified with the BCA Protein Assay Kit (Bio-Rad, United States). The sample was stored at −80°C.

### SDS-PAGE Separation

Twenty µg of proteins for each sample were mixed with 5X loading buffer and boiled for 5 min and were separated on 12.5% SDS-PAGE gel (constant current 14 mA, 90 min). Protein bands were visualized by Coomassie Blue R-250 staining.

### Filter-Aided Sample Preparation (FASP Digestion)

Two hundred μg of proteins for each sample were incorporated into 30 μl SDT buffer (4% SDS, 100 mm DTT, 150 mm Tris-HCl pH 8.0). The detergent, DTT, and other low-molecular-weight components were removed using UA buffer (8 M Urea, 150 mm Tris-HCl pH 8.0) by repeated ultrafiltration (Microcon units, 10 kD). Then, 100 μl iodoacetamide (100 mm IAA in UA buffer) was added to block the reduced cysteine residues, and the samples were incubated for 30 min in the darkness. The filters were washed with 100 μl UA buffer three times, and then, with 100 μl 100 mm TEAB (triethylamine borane) buffer twice. Finally, the protein suspensions were digested with 4 μg trypsin (Promega) in 40 μl TEAB buffer overnight at 37°C, and the resulting peptides were collected as a filtrate. The peptide content was estimated by UV light spectral density at 280 nm, using 1.1 of 0.1% (g/L) solution extinction coefficient which was calculated on the basis of the frequency of tryptophan and tyrosine in vertebrate proteins (Wiśniewski et al., 2009).

### Peptide Fractionation With High pH Reversed-Phase

Pierce high pH fractionation kit (reversed phase; Thermo Scientific) was used to fractionate TMT Tandem mass tag-labeled digest samples into 10 fractions by an increasing acetonitrile step-gradient elution, according to the instructions by the manufacturer.

### LC-MS/MS Analysis

Each fraction was injected for the nano LC-MS/MS analysis. The peptide mixture was loaded onto a reverse-phase trap column (Thermo Scientific Acclaim PepMap100, 100 μm*2 cm, nanoViper-C18connected to the C18 reversed-phase analytical column, Thermo Scientific Easy Column, 10 cm long, 75 μm inner diameter, 3 μm resin) in buffer A (0.1% Formic acid) and separated with a linear gradient of buffer B (84% acetonitrile and 0.1% Formic acid) at a flow rate of 300 nl/min controlled by IntelliFlow technology. The linear gradient was 1.5 h: 0–55% buffer B for 80 min, 55–100% buffer B for 5 min, 100% buffer B for 5 min. MS data were acquired using a data-dependent top 10 method dynamically choosing the most abundant precursor ions from the survey scan (300–1,800 m/z) for HCD fragmentation. An automatic gain control (AGC) target was set to 3e6, and maximum inject time to 10 ms. Dynamic exclusion duration was 40.0 s. Survey scans were acquired at a resolution of 70,000 at m/z 200 and resolution for HCD spectra was set to 17,500 at m/z 200 (TMT 6plex), 35,000 at m/z 200 (TMT 10plex), and isolation width was 2 m/z. The normalized collision energy was 30 eV and the underfill ratio, which specifies the minimum percentage of the target value likely to be reached at maximum fill time, was defined as 0.1%. The instrument was run with peptide recognition mode enabled.

### Protein Identification and Quantification

The MS/MS spectra were searched using the MASCOT engine (Matrix Science, London, United Kingdom; version2.2) embedded into Proteome Discoverer 1.4 (Thermo Fisher Scientific). Trypsin was chosen as the enzyme, allowing up to two missed cleavage sites. Carbamidomethylation (C), TMT 6-plex (N-term), and TMT 6-plex (lysine, K) were chosen as fixed modification. Oxidation (methionine, M) Was regarded as a variable modification. The peptide mass tolerances were set at 20 ppm for MS1 Spectra acquired, and the fragment mass tolerance for MS2 spectra was set to 0.1 Da. In this study, only rank one peptide and a false discovery rate (FDR) of ≤1% were accepted. The protein ratios were calculated as the median of only unique peptides of the protein. All peptide ratios were normalized to one by the median protein ratio. Comparisons of the protein identification and quantification results were done between each TDC media dose and the corresponding model groups. Significantly regulated proteins between experimental groups were determined based on their *p*-value (*p*-value < 0.05). Only proteins with more than 1.10-fold or less than 0.909-fold change compared. To control groups were considered differentially regulated.

### Bioinformatics Analysis

The protein sequences of differentially expressed proteins were in batches retrieved for GO mapping and annotation from the UniProtKB database (Release 2016_10) in FASTA format. The FASTA protein sequences of differentially changed proteins were blasted against the online Kyoto Encyclopedia of Genes and Genomes (KEGG) database (http://geneontology.org/) to retrieve their KOs and were subsequently mapped to pathways in KEGG12. The corresponding KEGG pathways were extracted. The acquired protein relative expression data was visualized as a tree heat map.

### Immunohistochemistry

Paraffin sections of kidney tissue (4 µm thick) were dewaxed, incubated with distilled water, incubated in 3% H_2_O_2_ humidification chamber for 10 min to block endogenous peroxidase, rinsed with PBS for 3 × 5 min, and repaired antigen in a microwave oven. After washing with PBS (3 × 5 min), the blocking buffer (10% BSA) was incubated for 1 h. Periostin antibody (1:300) was added, incubated at 4°C overnight or at 37°C for 1 h, washed with PBS (3 × 5 min). Biotinylated Goat Anti-Rabbit IgG was next added and incubated at 37°C for 40 min. DAB chromogenic agent was applied for about 4 min after washing with PBS (3 × 5 min).

Tissue slides were stained in Hematoxylin red for 40 s, sequentially with 1% hydrochloric acid ethanol differentiation, alkaline water blue, 70, 80, 95, 100% ethanol reverse dehydration, xylene clear liquid. Finally, slides were sealed with an automatic sealing machine. Protein expression in tissues was visualized and captured using an optical microscope (DP71, OLYMPUS, Japan). Staining areas were quantified using Image-Pro Plus 6.0 (National Institutes of Health, Bethesda, MD, United States) (Pei et al., 2013)

### Enzyme-Linked Immunosorbent Assay

Periostin (POSTN) ELISA kit (ZC-36167, Shanghai zcibio Technology Co., Ltd.) was used to determine the expression level of POSTN in renal tissue. The protein concentration of POSTN in the tissue was calculated by referring to the standard curve obtained from the kit with the standards in gradient concentrations.

### RNA Isolation and Quantitative Real-Time PCR Analysis

Total RNA samples from kidney tissues were isolated using TRIzol® reagent (Invitrogen, Thermo Fisher Scientific, Inc., Waltham, MA, United States) according to the manufacturer’s protocol. Subsequently, the complementary DNA (cDNA) was synthesized with a Verso cDNA synthesis kit (Thermo Fisher, United States), and real-time PCR was performed on a CFX96 (Bio-Rad, CA, United States). The Bestar ® Sybr Green qPCR Master Mix (DBI ® Bioscience, Ludwigshafen, Germany) was used in reverse transcription-quantitative real-time polymerase chain reaction (RT-qPCR) to quantify the mRNA expression levels of Periostin, TGF-β, and COL1A1 with glyceraldehyde 3-phosphate dehydrogenase (GAPDH) as an internal control. The data was calculated by melting curve and ‘Threshold Cycle’ (Ct) using CFX Manager Software. The primers are listed in Supplementary Table S1 and synthesized by Sangon Biotechnology Co., LTD (Shanghai, China).

### Protein Extraction and Western Blot Analysis

Kidney tissues were collected from the rats with different treatments, and total proteins were prepared with RIPA lysis buffer. Protein concentrations were measured by BCA from Solarbio, adjusted to 6.5 ug/μL, and stored at −20°C before Western Blot analysis.

The protein samples were subjected to SDS-PAGE gel and then transferred onto polyvinylidene difluoride (PVDF) membranes. After blocking by 5% skimmed milk, the membranes were incubated with primary antibodies against the specific anti-β-actin (1:6,000), anti-Periostin (1:1,000, Immunoway), and anti-α-SMA (1:1,000) for 1.5 h. Horseradish peroxidase (HRP) AffiniPure Goat Anti-Rabbit IgG (Beyotime) was added at a 1:10,000 dilution for 1 h at room temperature followed by TBST wash three times for 10 min each time. The immunoreactive protein bands were visualized by EasySee Western Blot Kit. The images were acquired using a Bio-Spectrum Gel Imaging System (UVP, United States).

### Culture of HEK293 Cells

Human embryonic kidney cell (HEK293) was purchased from the Cell-bank of the Chinese Academy of Sciences (Shanghai, China) and cultured in complete medium. The cells were placed in a constant temperature incubator at 37°C with 5% CO_2_. When reached 80–90% confluence, the culture medium was discarded, and cells were rinsed with PBS before adding trypsin for digestion. When the cells were detached, a complete culture medium was added to stop digestion and centrifuged at 1,000 rpm for 3 min.

### Immunofluorescence Quantification for Periostin Protein

HEK293 was exposed to Ang II (100 nm) for 24 h, then treated with different concentrations of TDC for 24 h, fixed in a 96-well plate with ice-cold ethanol at room temperature for 30 min. Cells were then treated with blocking solution (5% bovine serum albumin in phosphate buffer) for 30 min, incubated with primary anti-Periostin antibody (1:300), and incubated at 4°C overnight. The next day, after washing with PBST, the cells were incubated with Alexa Fluor 488 sheep anti-rabbit IgG (diluted 1:1,000) secondary antibody and Hoechst (1:300) for 2 h, and then fluorescence signals were quantitatively analyzed by a High Content Analysis System (Operetta, PerkinElmer).

### Sirius Red Assay for Collagen Content Determination

HEK293 cells were collected and inoculated into 96-well plates at a density of 1 × 10^4^/well. After 24h of adherent culture, the cells were changed to DMEM basic medium, and treated after 24h in the following conditions: normal control (untreated), model (5ng/ml TGFβ1), positive control (5ng/ml TGFβ1 + 5um SB431542) (Yang et al., 2020), Losartan (LST) (5ng/ml TGFβ1 + um LST), TDC high-dose (5ng/ml TGFβ1 + 0.4424mg/ml TDC), TDC medium dose (5ng/ml TGFβ1 + 0.1106mg/ml TDC), and TDC low dose (5ng/ml TGFβ1 +0.0276mg/ml TDC). After 48h of drug treatment, the supernatant was discarded, cells were fixed in frozen methanol at −20°C overnight and washed with PBS for three times. Picro Sirius Red (PSR) was added to each well and removed after 3h incubation in dark at room temperature. Unbound PSR was washed by 0.1% glacial acetic acid. After that, 200ul of 0.1mol/l NaOH was added to each well, and the absorbance was detected at 540nm after being placed in a shaker for 1h for complete dissolution.

### Statistical Analysis

All the results were expressed as mean ± SD. Statistical calculations were analyzed using GraphPad Prism seven software (GraphPad Software, Inc., La Jolla, CA, United States). To determine the statistical significance, a One-way analysis of variance followed by Tukey’s multiple comparison test was used. Kruskal-Wallis test was applied to compare the categorical data. A value of *p* < 0.05 was defined as a statistically significant difference.

## RESULTS

### Identification of Major Chemical Ingredients of TDC

**FIGURE 1:**
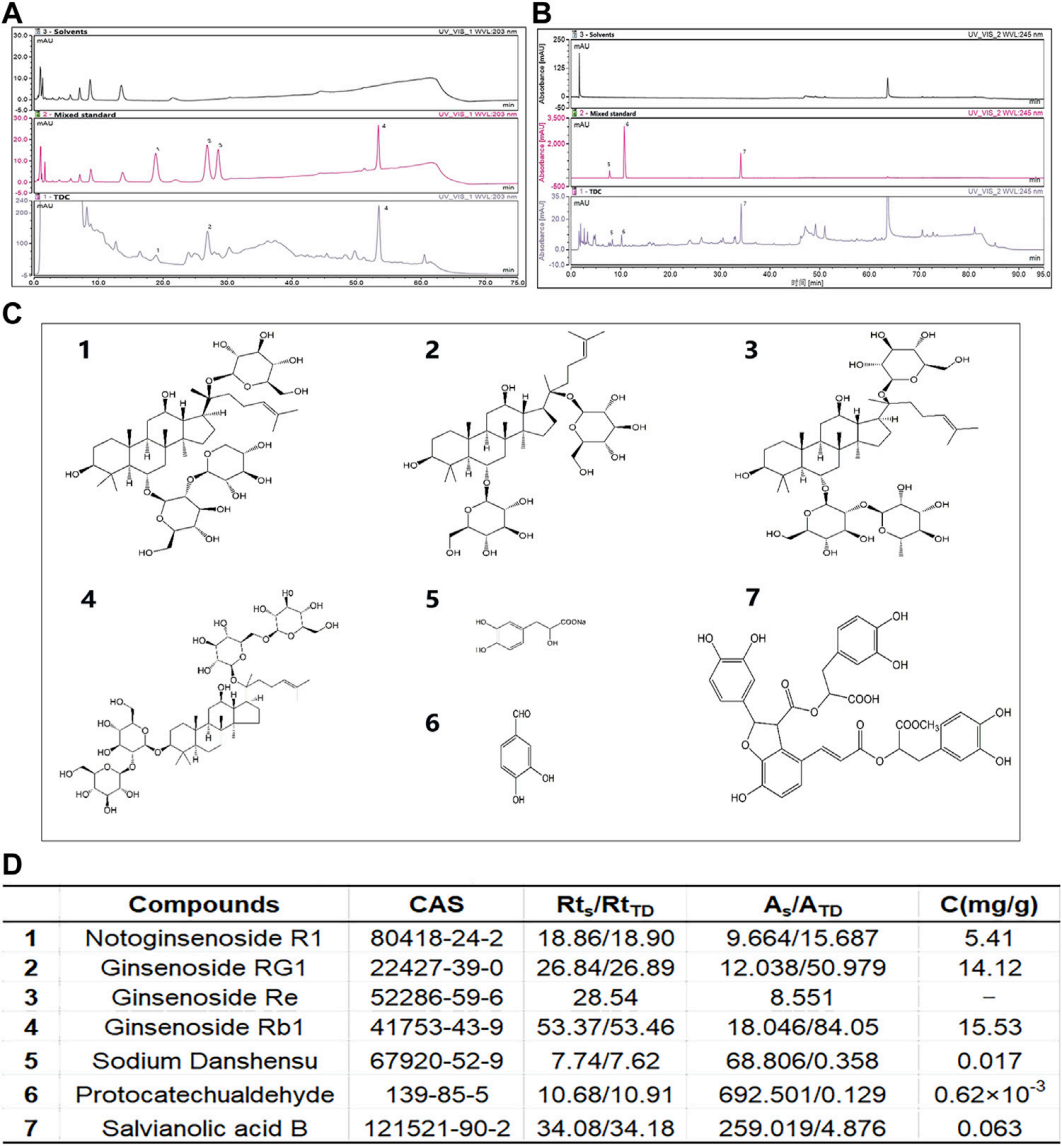
Identification of major chemical components in TDC **(A)** At 203 nm, solvents **(top panel)**, mixed standard **(middle panel)**, and water-soluble components of TDC **(bottom panel)** were determined by HPLC. The Acetonitrile-deionized aqueous solution was selected as the mobile phase **(B)** At 245 nm, solvents **(top panel)**, mixed standard **(middle panel)**, and water-soluble components of TDC **(bottom panel)** were determined by HPLC. Methanol-0.1% phosphoric acid aqueous solution was selected as the mobile phase **(C)** Chemical structures of the seven identified components in TDC **(D)** The name of the compound in TDC, retention time (Rt_S_ and Rt_TD_ represent the retention time of the standard component and TDC component respectively), peak area (A_S_ and A_TD_ represent the peak area of the standard component and TDC component respectively), and the content of each component in TDC. C (mg/g): Concentration in mg per g of total input. It is noted that because of the trace amount of compound 3 Ginsenoside Re, its quantitation was not successful.

We used HPLC to identify the major chemical components in the aqueous extracts of TDC. Double absorption wavelengths, 203 and 245 nm, were chosen for signal collection to maximize the information (Figures 1A,B). Through comparison with the standards, seven compounds were identified and quantified, namely Notoginsenoside R1, Ginsenoside RG1, Ginsenoside Re, Ginsenoside Rb1, Sodium Danshensu, Protocatechualdehyde, and Salvianolic acid B (Figures 1C,D).

### TDC Lowered Blood Pressures in SHR

**FIGURE 2:**
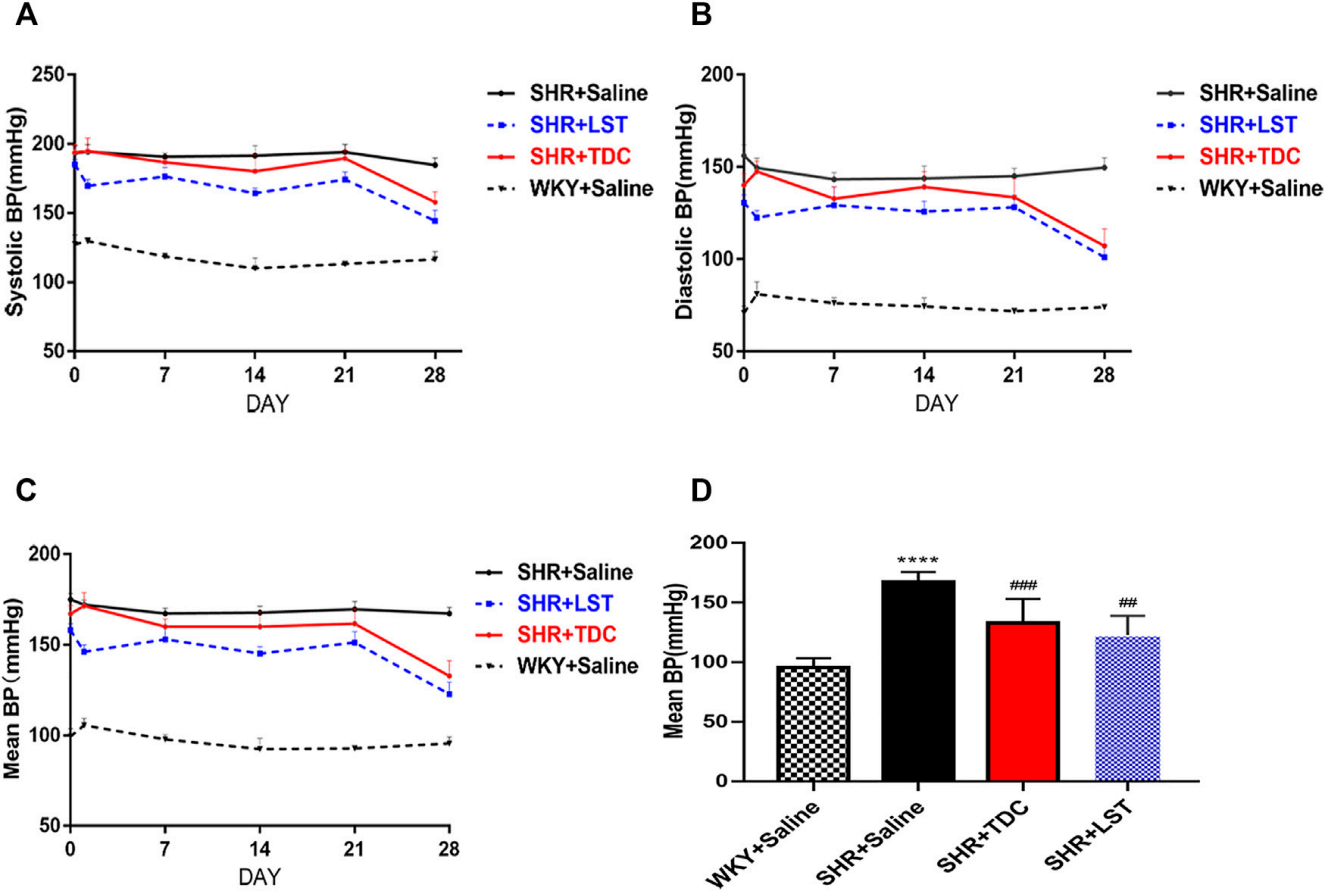
Effects of TDC on noninvasive blood pressure in SHR **(A–C)** Representative data plots of SBP, DBP, and MBP measurements, respectively, over 4 weeks using the noninvasive tail-cuff method in conscious rats **(D)** Bar graph comparison of the MBP in the four rat groups at day 28. Data are expressed as mean ± SD, *n* = 5–6. ^****^ *p* < 0.0001 vs. WKY rats; ^##^ *p* < 0.01, ^###^ *p* < 0.001 vs. SHR.

The effect of TDC on blood pressure regulation in SHR and age/gender-matched Wistar-Kyoto (WKY) rats was evaluated using losartan (LST) as a positive control drug (Yang et al., 2017). Results in Figure 2 showed that losartan (2 mg/ml) and a moderate dose of TDC (0.24 g/ml) had a significant antihypertensive effect compared with the saline control group (WKY), especially after 3 weeks of administration (Figures 2A–C). We quantified the mean pressure measured 4 weeks after administration, and the results showed that TDC was as effective as LST in reducing the mean blood pressure in SHR (Figure 2D).

### TDC Attenuated Hypertension-Induced Renal Damage and Fibrosis in SHR

**FIGURE 3:**
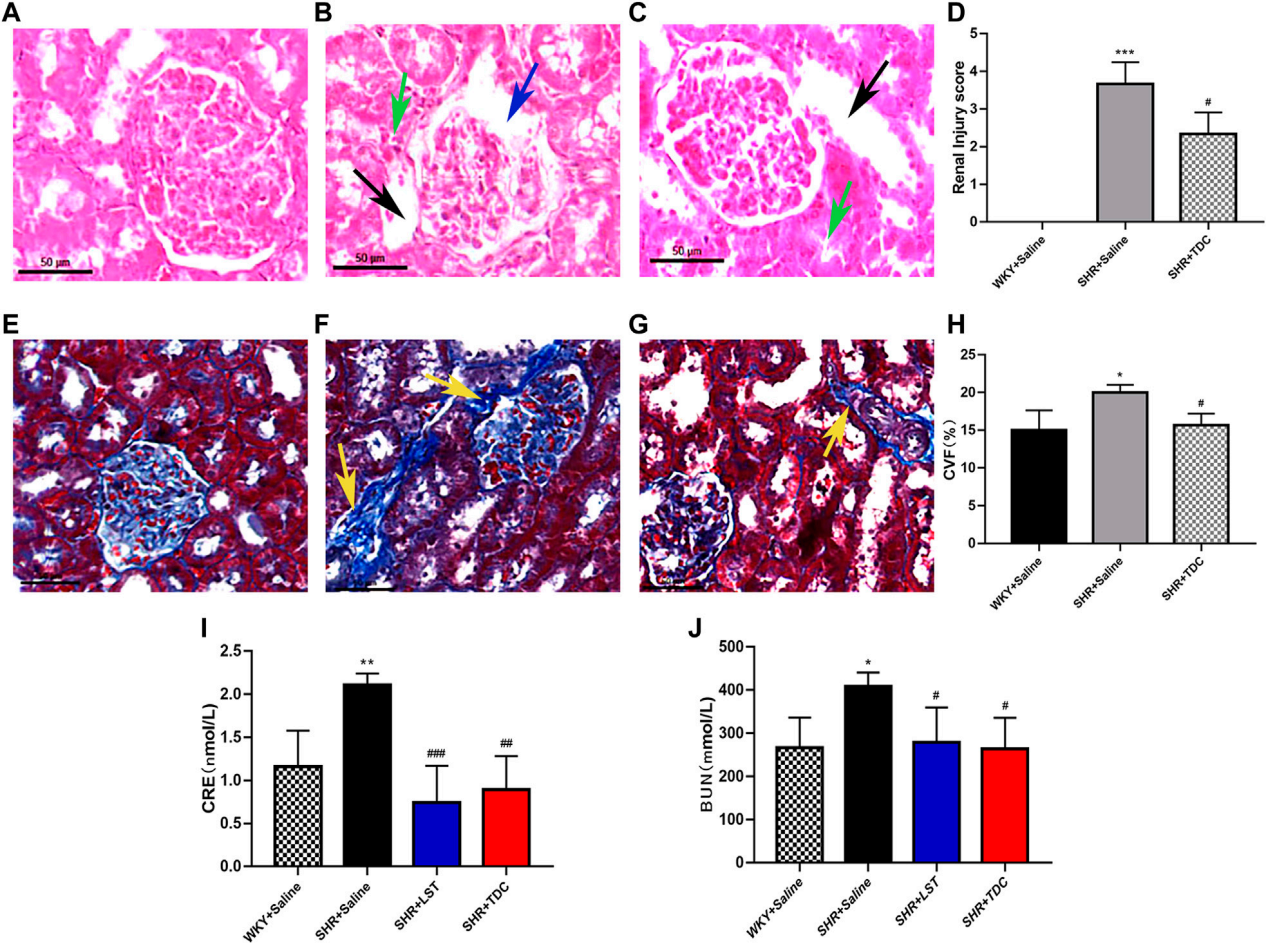
Effect of TDC on hypertension-induced renal injury in SHR **(A–J)** Panel **(A–C)**, Representative H and E staining photomicrographs of histopathological changes of renal cortex among different groups (×200 magnification) **(A)** Saline-treated WKY rats, showing normal architecture **(B)** Saline-treated SHR showing glomerular shrinkage and thickening of the basement membrane (blue arrows), with renal tubules dilation (black arrows) and tubular atrophy (green arrows) **(C)** TDC-treated SHR showing a significant reduction of glomerular shrinkage and thickening of basement membrane in the focal area **(D)** Bar graph comparison of the Renal Injury score in the three rat groups. Data are expressed as mean ± SD, *n* = 3. ^***^ *p* < 0.001 vs. WKY rats; ^#^ *p* < 0.05 vs. SHR (Saline). Panel **(E–G**), Representative Masson staining photomicrographs of histopathological changes of renal cortex among different groups (×200 magnification) **(E)** Saline-treated-WKY rats, showing normal architecture **(F)** Saline-treated SHR showing a significant increase in collagen fibers (yellow arrows) **(G)** TDCs-treated SHR showing obvious reduction of fibrosis in the focal area **(H)** Bar graph comparison of the Collagen volume fraction (CVF) levels in the three rat groups. Data are expressed as mean ± SD, *n* = 3. ^*^ *p* < 0.05 vs. WKY rats; ^#^ *p* < 0.05 vs. SHR (Saline). Panel **(I–J)**, Bar graph comparison of the levels of CRE and BUN in the four rat groups. Data are expressed as mean ± SD, *n* = 4–5. ^*^ *p* < 0.05 ^**^ *p* < 0.01 vs. WKY rats; ^#^ *p* < 0.05 ^##^ *p* < 0.01 ^###^ *p* < 0.001 vs. SHR.

At the organ level, H&E stain revealed histopathological changes in the renal cortex among different treatments (Figures 3A–D). Compared with that of WKY controls, SHR kidneys showed obvious contraction and thickening of the glomerular basement membrane (blue arrow), dilated tubules (black arrow), and shrank tubules (green arrow). TDC significantly prevented the glomerular contraction and basement membrane thickening in the lesion area of SHR kidneys after 4 weeks of treatment.

Masson staining showed that compared with the WKY control group, the degree of collagen fiber in the kidney of the SHR model group was significantly increased (Figures 3E–H, yellow arrow), whereas TDC reduced the fibrosis in the lesion after 4 weeks of treatment (Figures 3E–H).

CRE and BUN, the two important indicators of kidney function, were used to indirectly evaluate renal injury by reflecting the filtration capacity of the glomerulus (Sui et al., 2017). Compared with the WKY rat urine, the CRE and BUN levels of the SHR urine were clearly increased, whereas those of losartan- or TDC-treated SHR were significantly decreased after 4 weeks of administration (Figure 3I–J ).

### Identification of Differentially Expressed Proteins in the Kidney of SHR Induced by TDC

**FIGURE 4:**
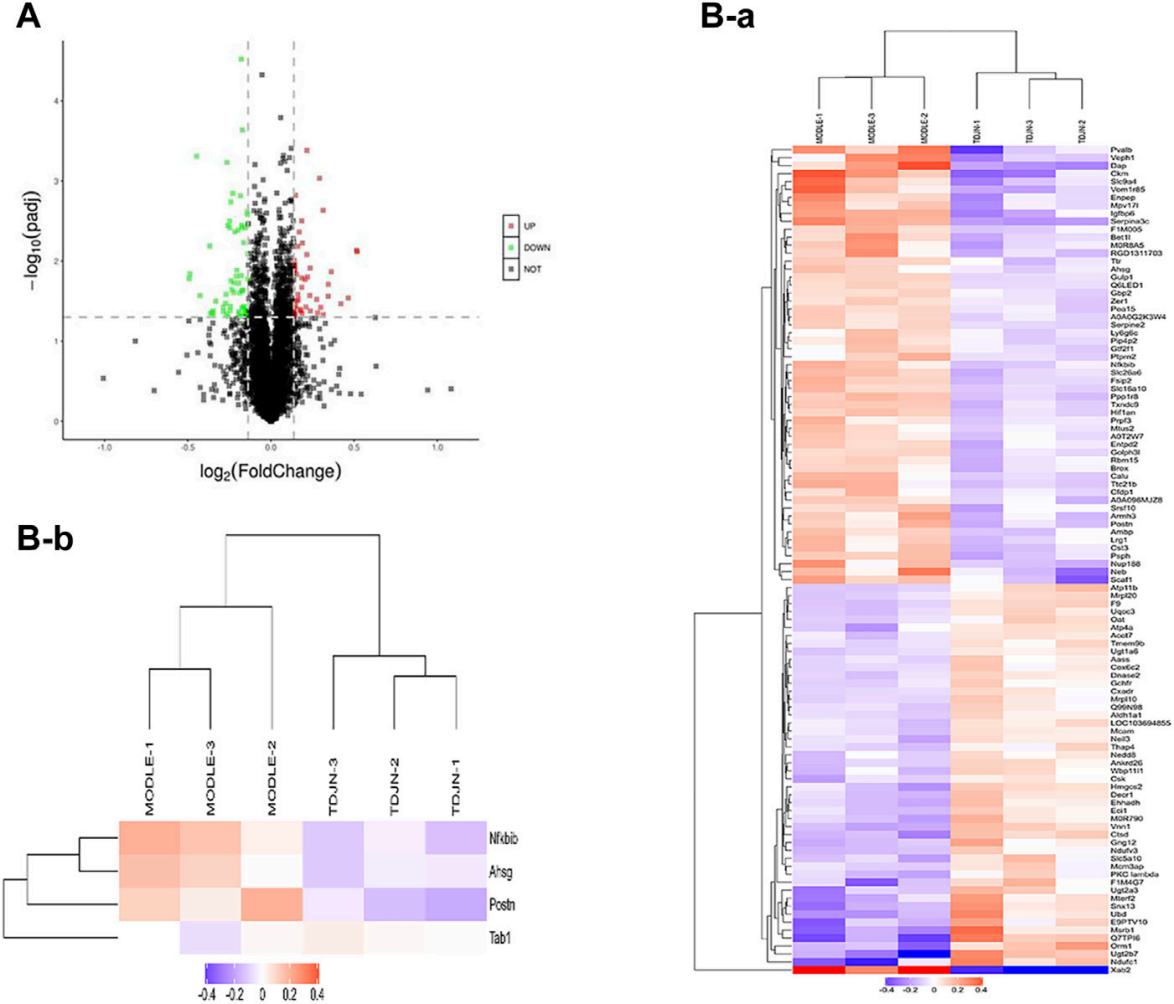
Pharmacoproteomics of SHR kidneys after TDC treatment **(A)** Volcano plot illustrating proteins with different abundance in model and TDC treatment groups. The Log2 ratios of protein intensities of TDC treatment to the Control group were plotted against the negative Log10 *p* values. Red points represent upregulated proteins while green points represent downregulated proteins in the TDC treatment group (fold change >1.1, *p*-value <0.05), and gray points represent unchanged proteins **(B)** Clustering of the differentially expressed proteins. The changed proteins were assigned to two clusters (a, b) by fuzzy c-means (FCM) clustering algorithm. The *y*-axis is log10 transformed and normalized. Each trace was colored according to its membership value in the corresponding cluster (referred to membership color bar).

In order to reveal the potential molecular mechanism of TDC in renal protection of rats with essential hypertension, TMT-based quantitative proteomics was performed to conduct a quantitative analysis of proteins extracted from the kidney of the model and TDC-treated SHR after 4 weeks of administration. A total of 5,688 proteins were identified by one or more unique peptides. With a threshold of fold change >1.10 or <0.91, *p*-value < 0.05, a total of 104 differentially expressed proteins in the renal tissues were detected between TDC-treated and the model groups. Among them, the expression level of 48 proteins was significantly increased with the fold change exceeding 1.10, while the expression level of 56 proteins was significantly decreased with the fold change less than 0.91, as shown in a volcano diagram (Figure 4A). The changed proteins were assigned to two clusters (Figures 4B) by fuzzy c-means (FCM) clustering algorithm. The Y-axis was log10 transformed and normalized. Each trace was colored according to its membership value in the corresponding cluster (referred to membership color bar). Information on the differentially expressed proteins is shown in Supplementary Table S2. By mining the literature and protein databases, periostin was identified as a primary candidate since it has been shown to mediate inflammation and fibrosis in a variety of organs and periostin inhibition in animal models of multiple diseases effectively prevents pathological progression (Oka et al., 2007; Shimazaki et al., 2008; Sidhu et al., 2010; Zhou et al., 2010; Lorts et al., 2012; Naik et al., 2012; Uchida et al., 2012; Yang et al., 2012; Ozdemir et al., 2014; Nakama et al., 2015; Sugiyama et al., 2016). Periostin was indeed one of the most significantly reduced proteins by TDC in SHR kidney (reduced 0.85-fold, *p* = 0.017). Therefore, periostin was further investigated *in vivo* and *in vitro* to evaluate its effect on renal pathological progression in SHR or renal cells.

### TDC Protected Rat From Hypertension-Induced Kidney Fibrosis Through Inhibiting the Periostin-Mediated TGF-β Pathway

**FIGURE 5:**
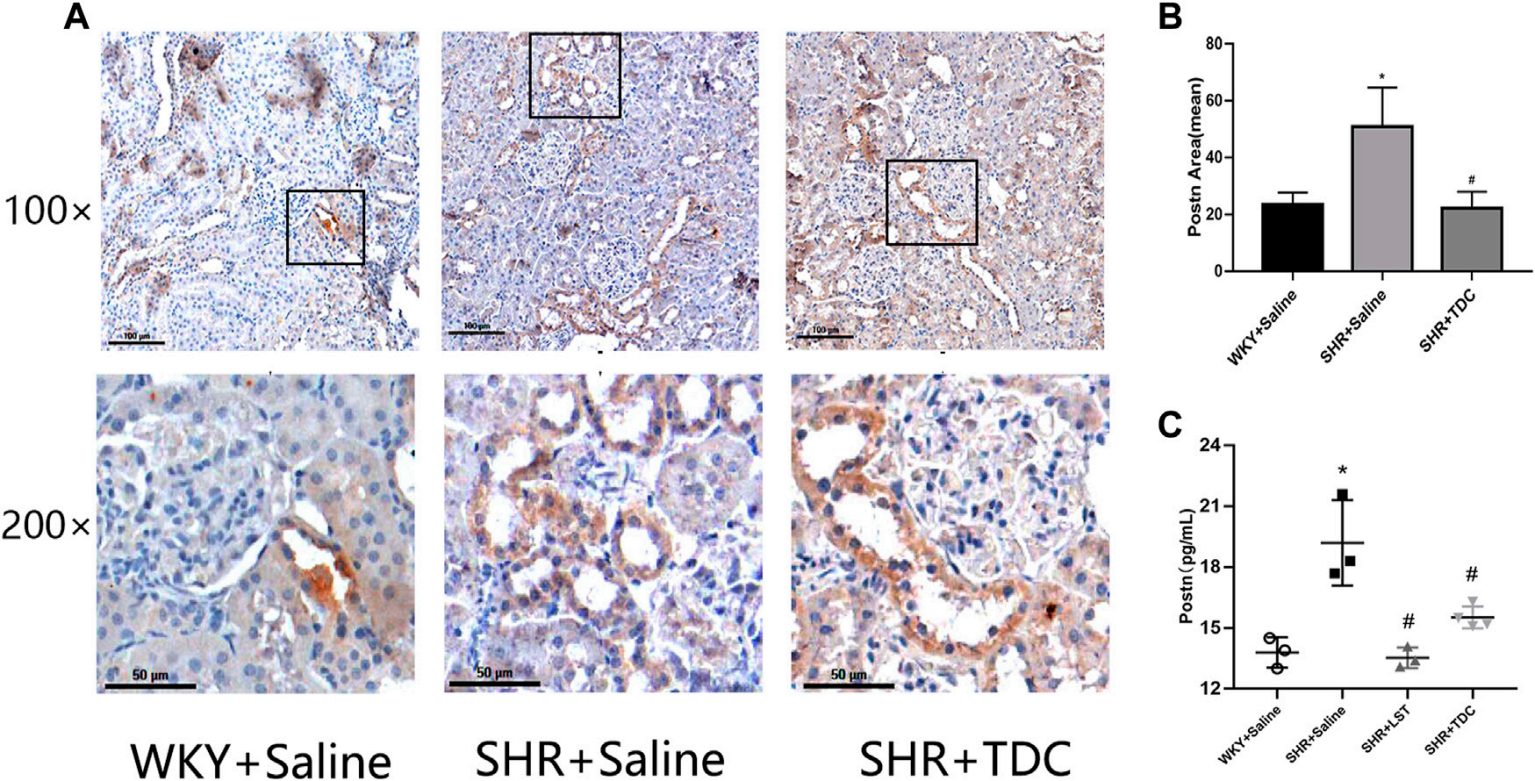
Effects of TDC on periostin protein expression in SHR kidney *in situ*. After 4 weeks of treatment with TDC, periostin protein expression in renal tissues was detected by **(A, B)** immunohistochemistry and **(C)** ELISA **(A)** Representative immunohistochemical staining images for the kidney tissue section of SHR and WKY rats at ×100 **(top)** and ×200 **(bottom)** magnifications. Periostin protein was stained in brown **(B)** Bar graph quantification of periostin expression **(C)** Quantification of ELISA for periostin expression in renal tissue supernatants. Data are expressed as mean ± SD, *n* = 3. ^*^ *p* < 0.05 vs. WKY rats; ^#^ *p* < 0.05 vs. SHR.

Immunohistochemical (IHC) analysis showed that periostin was reduced by treatment with TDC in SHR as compared to WKY rats after 4 weeks (Figures 5A,B). ELISA was used to detect the expression of periostin in different groups of kidney tissues. The results showed that compared with the SHR model group, the levels of periostin in both LST and TDC treatment groups were significantly reduced (Figure 5C).

**FIGURE 6:**
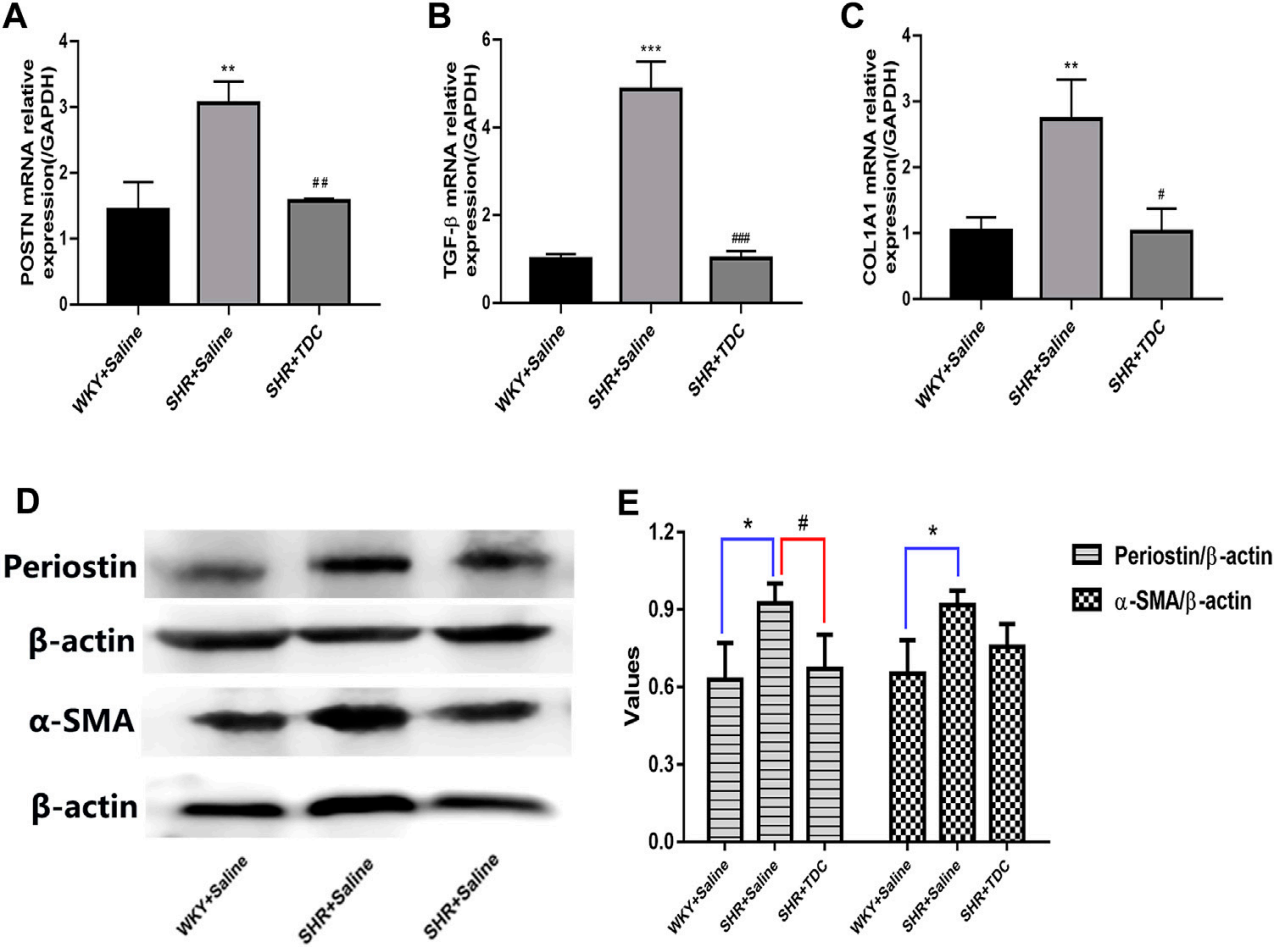
Effect of TDC on periostin and TGF-β-mediated fibrosis pathways **(A–C)** mRNA expressions of periostin **(A)**, TGF-β **(B)** and COL1A1 **(C)** were determined with real-time qPCR and data were normalized to that of GAPDH **(D–E)** protein expression of periostin and *α*-SMA were assessed by Western blot **(D)** Representative images and **(E)** Bar graph quantification of the WB data. Data are expressed as mean ± SD, *n* = 3. ^*^ *p* < 0.05 ^**^ *p* < 0.01, ^***^ *p* < 0.001 vs. WKY rats; ^#^ *p* < 0.05, ^##^ *p* < 0.01, ^###^ *p* < 0.001 vs. SHR.

Quantitative RT-PCR results further verified the anti-inflammatory and anti-fibrosis activities of TDC. The mRNA expressions of POSTN (periostin), TGF-β and COL1A1 in the SHR model group were increased after 4 weeks compared with those of the WKY group, while those in the TDC treatment group were decreased significantly (Figures 6A–C). Western blotting was then used to confirm the results of the TMT-based quantitative proteomic analysis. As shown in Figures 6D,E, periostin and α-SMA expressions in SHR kidney were increased compared with that in WKY rats, whereas both levels were decreased significantly after 4 weeks of TDC administration.

### TDC Decreased Periostin Expression *In Vitro* in Angiotensin II-Treated HEK293 Cells

**FIGURE 7:**
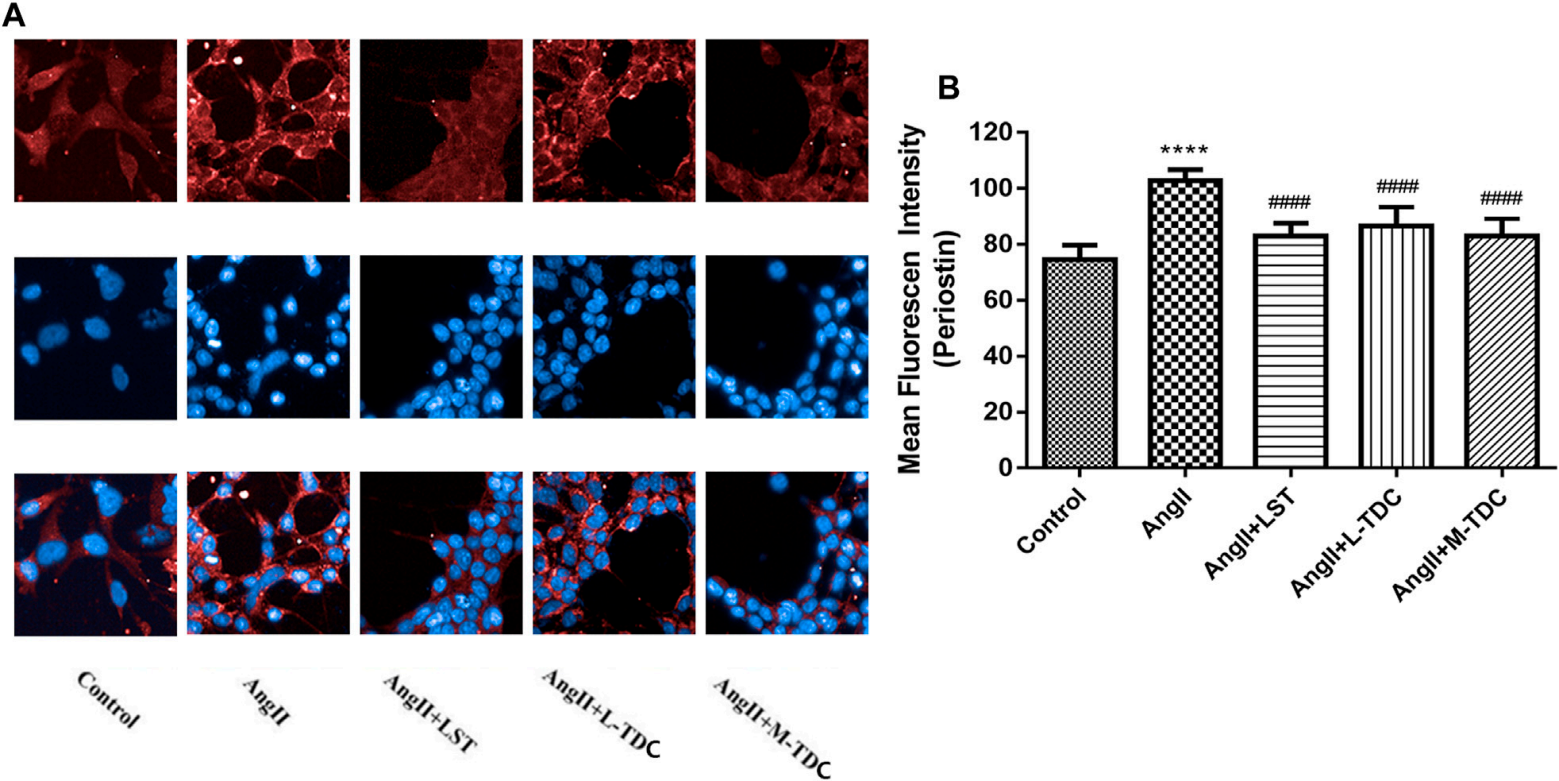
Effect of TDC on periostin expression in Ang II-treated HEK293 cells **(A)** Representative immunofluorescence stain images (×200 magnification) of anti-periostin antibody (top, red), nucleus (middle, blue), and merge (bottom). LST: positive-control drug losartan (1 μmol/l); L-TDC: low dose of TDC (0.0138 mg/ml); M-TDC: medium dose of TDC (0.0553 mg/ml) **(B)** Quantitation of IF results from three independent experiments. Data are expressed as mean ± SD, *n* = ,3. ^###^ *p*,<,0.001 vs. Control; ^***^ *p*,<,0.001, vs. Ang Ⅱ-induced HEK293 cells.

To investigate the effect of TDC on periostin expression in hypertensive renal cells *in vitro*, HEK293 human renal cells were treated with Ang II. Immunofluorescence (IF) analysis showed that compared with untreated control cells, Ang II significantly increased periostin expression in HEK293 cells. Anti-hypertensive drug losartan (1 µm) decreased the expression of periostin. Similarly, medium (0.0553 mg/ml) and low dos es (0.0138 mg/ml) of TDC reduced the expression of periostin in Ang II-treated cells (Figures 7A,B).

### TDC Lowered TGFβ1-Induced Collagen Deposition in HEK293 Cells

**FIGURE 8:**
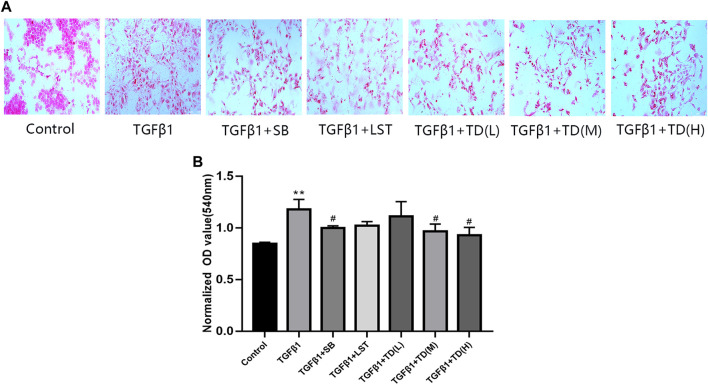
Effect of TDC on collagen deposition of TGF-β1-treated HEK293 cells **(A)** Representative image of Sirius red staining (×200 magnification), with red representing collagen deposition. SB: positive-control drug TGF-β1 inhibitor SB431542 (5 μmol/l); LST: losartan (1 μmol/l); L-TDC: low dose of TDC (0.0138 mg/ml); M-TDC: medium dose of TDC (0.0553 mg/ml); H-TDC: high dose of TDC (0.2212 mg/ml) **(B)** Quantitation of Sirius red staining results from three independent experiments. Data are expressed as mean ± SD, *n* = 3. ^**^ *p* < 0.01, vs. Control; ^#^ *p* < 0.05, vs. TGF-β1 induced HEK293 cells.

To investigate the *in vitro* effect of TDC on renal fibrosis, a collagen deposition assay was performed in HEK293 cells. Compared with the blank control, TGF-β1 induced collagen deposition by about 1.4 folds, and TGF-β1 inhibitor SB431542 (5 μmol/l) reduced it (Supplementary Table S3 and Figure 8). Both medium dose (0.0553 mg/ml) and high dose (0.2212 mg/ml) of TDC effectively inhibited collagen deposition. However, there was no significant change was observed in losartan (1 µm) and TDC low-dose (0.0138 mg/ml)-treated cells (Supplementary Table S3 and Figures 8A,B).

## Discussion

High blood pressure affects more than 1.2 billion people worldwide and has become the most serious and expensive public health problem. Its adverse effects include accelerating the development of terminal organ diseases, such as the heart, brain, and kidneys, among which the progressive loss of renal function is particularly important and is the main cause of “end-stage renal disease” (ESRD). Hypertensive nephropathy is a disease with primary hypertension as the main cause, which in turn leads to renal damage, arteriolosclerosis, nephron atrophy, and renal dysfunction (Mennuni et al., 2014). The effects of BP-lowering drugs with different antihypertensive mechanisms may be cumulative, synergistic, or complementary in combination (Mahmud and Feely, 1979; Law et al., 2003; Kizilirmak et al., 2013; Chow et al., 2017; Webster et al., 2018), which is conducive to taking into account various risk factors and complications of patients, improving patient compliance and quality of life, and other advantages. Therefore, blood pressure-lowering drugs are the preferred treatment regimens recommended by antihypertensive guidelines of various countries in the world. However, the mechanism of hypertension is complex, and it is often accompanied by other cardiovascular and cerebrovascular diseases and even other visceral lesions. At present, the commonly used antihypertensive drugs have different clinical indications and adverse reactions.

The study of Chinese herbology for treating hypertension is the most active area of research within TCM and integrative medicine in China. Over the past 40 years, significant progress has been made from theory and experiments, in the clinical fields based on the inheritance and innovation of thoughts in Traditional Chinese Medicine, to clarify the treatment regulations and principles of hypertension (Lyu et al., 2016). Several studies have reported the effects of the protective effects of TCM treatment on renal damage caused by hypertension. According to the Guidelines for diagnosis and treatment of hypertension in traditional Chinese medicine (Anthony et al., 2015), clinical hypertension is categorized into Liver-Fire Ascending, Dampness Phlegm Accumulation syndromes, blood stasis internal resistance, and Yin Deficiency-Yang Hyperactivity. The principle of TCM treatment of hypertension is mainly to reinforce the insufficiency, and reduce excessiveness, so as to achieve a balance between Yin and Yang. Therefore, existing antihypertensive Chinese patent medicines are mainly to clear heat and purge fire, calm liver latent heat Yang, promoting blood circulation and removing blood stasis, and nourishing liver and kidney (Lyu et al., 2016). TDC is a compound TCM composed of 10 Chinese herbs. Studies have shown that *Uncaria rhynchophylla Miq* from the Rubiaceae family is a significant plant source of active alkaloids, with anti-hypertensive, sedative, anti-Alzheimer’s disease, anti-drug addiction, and anti-inflammatory effects (Qin et al., 2020). *Redix Salvia miltiorrhizae* is a traditional medicinal plant, which is widely used in Asian countries due to its pharmacological effects. Studies have shown that one of its active ingredients, *Redix Salvia miltiorrhiza*, has obvious anti-inflammatory and cardiovascular protective effects (Hu et al., 2020). Another active ingredient, Protocatechualdehyde, is a major component of salvia miltiorrhiza that reduces cardiovascular ischemic injury by inhibiting the ER stress-related PERK, IRE1α, and ATF6α signaling pathways (Wan et al., 2020). *Panax pseudoginseng* Wall is rich in saponins, volatile oil, and polyacetylenes, which has a good protective effect on the cerebrovascular system (Liu G. et al., 2020). Recently, some well-controlled clinical trials have proved the cardiovascular protection of *Astragalus mongholicus Bunge* and its extraction (Piao and Liang, 2014; Li et al., 2018). *Astragalus* armour glycosides (ASG) is one of the active ingredients with high content in the *Astragalus membranaceus*. Studies indicated that ASG could inhibit cardiac fibrosis *via* the TRPM7 channel (Wei et al., 2020). Therefore, TDC was formulated in accordance with the principle of simultaneous treatment, for subordinate symptoms, in order to reduce the target organ damage caused by hypertension while reducing hypertension. Despite its wide availability in hospitals and pharmacies in China, the preclinical research on TDC is limited to pharmaceutics, quality control standard, pharmacodynamics, acute and long-term toxicities, and stability. It is important and critical to understanding more about the chemical basis and mechanisms of TDC's anti-hypertensive effects.

The main finding of this study was that TDC significantly reduced BP (diastolic, systolic, and mean blood pressure) (Figures 2A–C), reduced urinary creatinine and urea nitrogen (biomarkers of renal injury) (Figures 3I,J), and prevented the glomerular contraction and basement membrane thickening (Figures 3A–C) in SHR. This confirmed and extended its clinical efficacy. At the same time, it inhibited fibrosis in the renal lesion area (Figures 3E–G). Proteomic analysis revealed that TDC blocks periostin, which plays a crucial role in the progression of renal diseases through the regulation of TGF-β/Smad signaling. *In vitro* study confirmed that TDC decreased periostin expression in AngII-treated HEK293 human renal cells (Figures 7A,B) and lowered TGFβ1-induced collagen deposition in HEK293 (Figures 8A,B).

Seven major compounds were identified and quantified from the water-soluble extracts of TDC by HPLC. The results showed that three components, Sodium Danshensu, Protocatechualdehyde, and Salvianolic acid B, were isolated and identified by gradient elution using methanol-0.1% phosphoric acid system as mobile phase at 245 nm, and the peak shape and separation degree of each chromatographic peak were relatively well. Notoginsenoside R1, Ginsenoside RG1, Ginsenoside Rb1 were isolated and identified by gradient elution, using acetonitrile - aqueous solution system as mobile phase at 203 nm. Ginsenoside Rg1 and Ginsenoside Re were isolated in the mixed standard, but in the TDC samples, the peak area of Ginsenoside Re was small, and it was not effectively separated from Ginsenoside Rg1. The reasons for this may be that the content of Ginsenoside Re in TDC was low, and the gradient elution flow phase ratio used in the separation was not optimal. According to the analysis results, it is clear that most of the characteristic peaks of TDC are from two herbs, *Redix Salvia miltiorrhizae,* and *Panax notoginseng*. Through quantitative comparison, it can be seen that the contents of Rg1 and Rb1 in *Panax notoginseng* are higher (Figure 1D). However, components from *Uncaria rhynchophylla Miq*, *Prunella Vulgaris L*, or *Astragalus mongholicus Bunge* have yet to be identified. One reason may be that in the ultrasonic process of an aqueous solution, few or no components in the medicinal materials exist due to their more lipid-soluble nature. In addition, due to the limitation of detection sensitivity of the instrument and lack of proper reference substance, it is difficult to identify the components. Therefore, this initial experiment cannot fully reflect the chemical composition of TDC. In our study, the identification of chemical components of TDC by the HPLC method is only based on water-soluble components (Figure 1). Furthermore, we establish the fingerprint of the lipid-soluble components of TDC so as to provide a certain reference for the comprehensive evaluation of the compounds.

Most of the seven compounds identified in our chemical analysis have been shown to have anti-hypertensive activities in various animal models, for example, Notoginsenoside R1 (Yang et al., 2015), Ginsenoside RG1 (Chen et al., 2012; Pan et al., 2012; Li et al., 2013), Ginsenoside Rb1 (Wang et al., 2015; Jiang et al., 2017; Wang et al., 2020), Sodium Danshensu (Tang et al., 2011; Zhang et al., 2016; Zhang et al., 2018; Liu H. et al., 2020), and Salvianolic acid B (Fu et al., 2012; Xu et al., 2012; Zhao et al., 2012; Du et al., 2014; Ling et al., 2017). Interestingly, Ginsenoside RG1 has also been shown to have a protective effect for organ damage in SHR (Chen et al., 2012). Therefore, the anti-hypertensive and renal protective effects of TDC revealed in our study could be attributed to those of the individual compounds, or from other unidentified compounds, or the combination of them. However, future studies are needed to provide direct evidence for these possibilities.

For an experimental study of herbal medicine, choosing the proper *in vivo* and *in vitro* doses are always a concerning issue. The clinical dosage of TDC was 5 pills (0.4 g per pill) each time, three times daily. To compare the efficacy of TDC on blood pressure regulation in SHR and WKY rats, Losartan (LST, 50 mg/tablet twice daily for clinical use) was used as a positive control drug. In this study, rats were given LST (10 mg/kg) or TDC once a day by intragastric administration. According to human and rat dose conversion formula and setting a 0.5 ml drug volume for every 100 g body weight of rats, it is equivalent to give LST at 2 mg/ml, TDC at a low dose (0.12 g/ml), medium dose (0.24 g/ml) and high dose (0.48 g/ml), respectively. Our pilot study showed that the anti-hypertensive effect of medium dose (0.24 g/ml) was more prominent, and the rats with this concentration had a high acceptance degree by gavage administration (data not shown). Therefore, the medium dose was chosen for the rest of the *in vivo* study. For the *in vitro* study, a pilot cell viability test was performed with a serial range of TDC dilutions. The determined doses used in final experiments were low at 0.0138 mg/ml, medium at 0.0553 mg/ml and high at 0.2212 mg/ml, respectively.

In our study, the angiotensin II receptor blocker losartan is shown to reduces periostin expression even more dramatically than TDC (Figure 7) *in vitro*, suggesting that losartan has a better kidney protection effect. This is consistent with the previous findings by others. For example, losartan alleviates renal fibrosis in rats with nephrectomy by down-regulating HIF-1alpha and up-regulating MMP-1/TIMP-1 or through inhibition of ER stress *via* up-regulation of SIRP1, followed by induction of HO-1 and thioredoxin (He et al., 2014; Kim et al., 2017); it attenuates renal interstitial fibrosis and tubular cell apoptosis in a rat model of obstructive nephropathy (Song et al., 2019) and accelerates the repair process of renal fibrosis in UUO mouse after the surgical recanalization by upregulating the Tregs expression (He et al., 2021). Most recently, losartan is shown to prevent bladder fibrosis and protects renal function in rat with neurogenic paralysis bladder (Norris et al., 2007).

**FIGURE 9:**
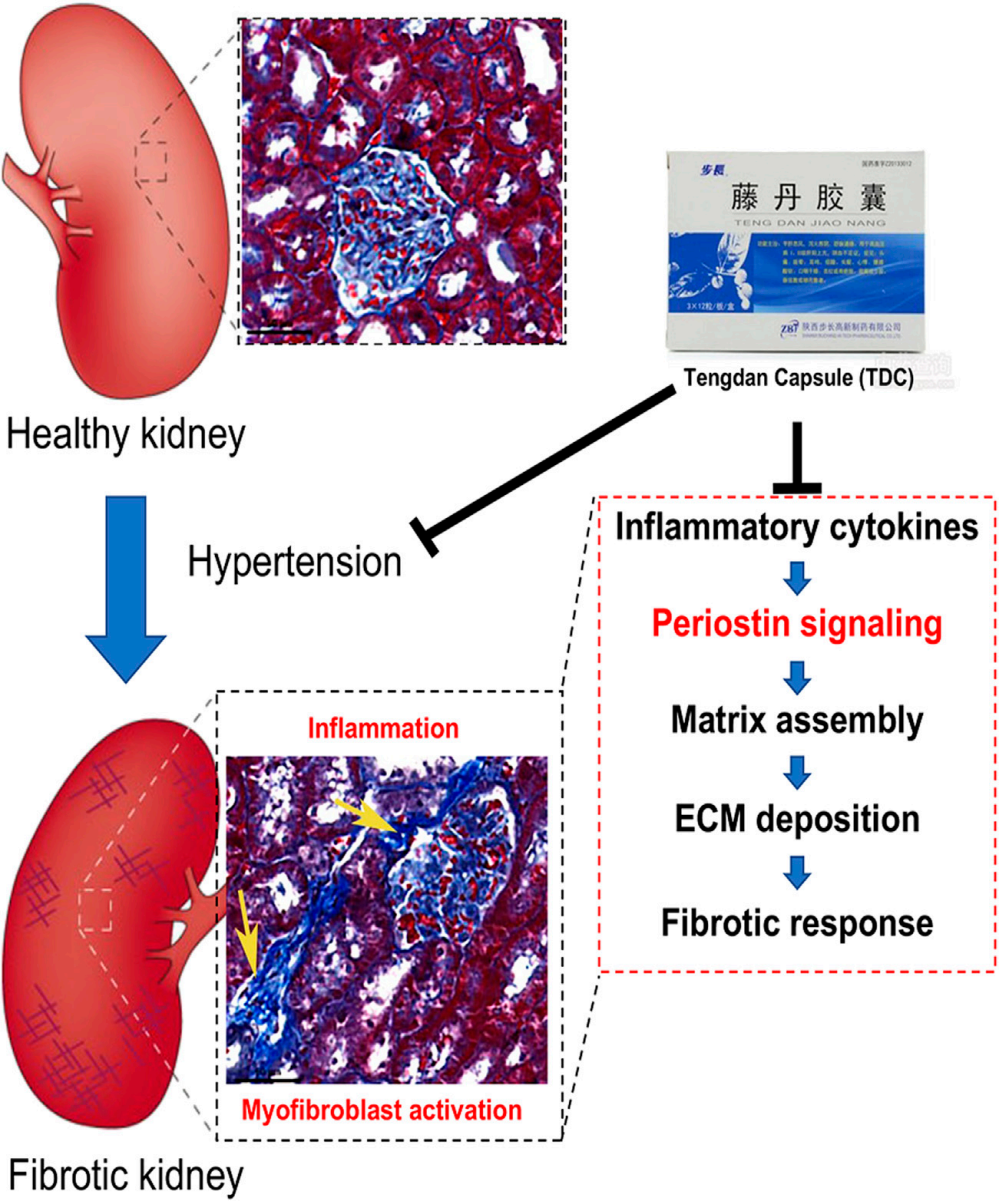
Schematic diagram of how Tengdan Capsule (TDC) prevents progression of hypertensive renal damage *via* periostin-mediated pathway. Hypertension causes renal inflammation and myofibroblast activation. Different cytokines and growth factors upregulate periostin expression. Periostin interacts with its integrin receptors on the cell surface, inducing inflammation and matrix assembly and promoting cell adhesion, migration, and proliferation. TDC reduces blood pressure as well as blocks these pathways that amplify the inflammatory and fibrotic responses.

The pathological study showed that TDC treatment on SHR had significant inhibitory effects on glomerular contraction, basement membrane thickening, and fibrosis in the lesion areas. In addition, the elevated expressions of TGF-β, COL1A1, and α-SMA in SHR were all decreased to different degrees by TDC treatment. Quantitative tandem mass tag (TMT) proteomics was applied on kidneys of the model (SHR) and treatment (TDC for 4 weeks) rats, revealing “POSTN” (periostin) as one of the differentially expressed proteins. Periostin is a 90 kDa matricellular protein that is highly expressed in bone and tooth tissues. Like other such proteins, periostin expression is high during development but is limited in adult tissues. Its expression level is also significantly upregulated under injury and wound healing conditions (Prakoura and Chatziantoniou, 2017). Studies have shown that periostin interacts with several proteins of the extracellular matrix such as Notch-1, collagen-1, BMP-1, tendinoprotein C, and various cell surface integrins through its different domains (Gillan et al., 2002; Snider et al., 2008; Kii et al., 2010; Maruhashi et al., 2010; Tanabe et al., 2010), which promote the formation of collagen fibers and enhance the strength performance of extracellular matrix (Coffman, 2011). Through these interactions, periostin mediates signals from the extracellular and intracellular environment, thereby regulating extracellular matrix assembly and cell adhesion, migration, and differentiation (Prakoura and Chatziantoniou, 2017) (Figure 9). Consistently, we found that Ang ǁ induced high expression of periostin in human kidney HEK293 cells *in vitro*, which were significantly decreased by TDC (Figures 7A,B). Our results also showed that compared with the control, TGF-β1 induced collagen deposition in HEK293 cells increased, while TDC effectively inhibited the collagen deposition. This protection may be caused by a dual mechanism: reduced inflammatory flow and reduced TGF-β signaling pathway. Recent studies have shown that immune system cells are involved in the pathogenesis of hypertension through their role in the kidney, blood vessels, and brain, so immune regulation may be a new approach to reduce blood pressure and to limit hypertension-induced organ damage (Schreiner and Kohan, 1990; Crowley et al., 2011; Crowley and Jeffs, 2016). Transforming growth factor-β (TGF-β) is a key driver of renal fibrosis, especially when activating the renin–Ang system (RAS), which is the main cause of hypertension (Kagami et al., 1994; Wei et al., 2013). TGF-β induces renal fibrosis by increasing the deposition of extracellular matrix proteins and inhibiting the activity of matrix metalloproteinases. One of the major and Important findings of this study was that the periostin pathway could be a target for the treatment of hypertensive renal damage. However, considering the complexity and redundancy of the TGF-β downstream fibrosis signaling pathway, a large number of experiments are still needed for verification (Rennenberg et al., 2010). Although these results suggest that periostin may serve as a new biomarker for the progression of renal disease, its role in the development of kidney disease is not fully clear. In the follow-up study, complementary methods such as knocking out or inhibiting periostin expression should be used to further validate this hypothesis.

Our proteomics study identified 104 differentially expressed proteins with or without TDC treatment in SHR. In addition to periostin, literature retrieval and PCR verification were performed for other proteins that might be related to hypertension, such as Ahsg, Cst3, and Lrg1. Ahsg is a plasma glycoprotein secreted mainly by the liver, which is associated with atherosclerotic calcification, insulin resistance, cardiovascular disease, and especially ischemic stroke (Weikert et al., 2008; Laughlin et al., 2012; Jiménez et al., 2014; Häring, 2016; Zhao et al., 2020). The results of Zhao et al. suggested that the level of Cst3 in circulation was upregulated in patients with hypertension and coronary artery disease, and Cst3 could serve as a marker to predict the occurrence of coronary artery disease in patients with hypertension (Watson et al., 2011). Watson et al. recently showed that Lrg was identified as a serum biomarker that accurately identifies patients with heart failure (Perez-Riverol et al., 2019). We intend to conduct further research on these differentially expressed genes and explore their possible roles in the target organ damage caused by hypertension.

In this study, integrated medicinal chemistry, proteomics, and traditional pharmacological methods are used to evaluate the efficacy and mechanism of water-soluble active components of TDC for hypertensive renal injury. *In vitro*, AngⅡ and TGFβ1-induced renal damage of HEK293 human renal cells are used to confirm that TDC significantly reduces the hypertensive renal damage. More importantly, we demonstrate that TDC blocking periostin by regulating TGF-β/Smad signaling may be a promising strategy for treating a hypertensive renal injury. It also provides a new way of thinking for anti-hypertension therapy by a multi-component medicine that targeting not only blood pressure-lowering but also organ protection.

## Data Availability

The datasets presented in this study can be found in online repositories. The name of the repository and accession number can be found below: ProteomeXchange, http://www.proteomexchange.org/, PXD024102.

## Glossary

TDC: Tengdan capsule
HPLC: High performance liquid chromatography
POSTN: Periostin
SHR: Spontaneously hypertensive rats
WKY: Wistar-Kyoto
BP: blood pressure
CRE: Creatinine
BUN: Blood urea nitrogen
H&E: Hematoxylin and Eosin
qRT-PCR: Quantitative reverse transcription polymerase chain reaction
RAAS: Renin-angiotensin-aldosterone system
TCM: Traditional Chinese medicine
ECM: extra cellular matrix
EMT: epithelial-to-mesenchymal transition
TGF-β: Transforming growth factor-β
TMT: Tandem mass tag
AGC: Automatic gain control
FDR: false discovery rate
KEGG: Kyoto Encyclopedia of Genes and Genomes
PVDF: polyvinylidene difluoride
HRP: Horseradish peroxidase
FCM: fuzzy c-means
IPA: Ingenuity ® Pathway Analysis
BCA: Bicinchoninic acid assay
LST: losartan
IHC: Immunohistochemical
IF: Immunofluorescence
L/M/H: Low/medium/high dose
SDT: 4%SDS,100mMTris-HCl, 1 mM DTT, pH7.6
TEAB: triethylamine borane
TCA: Trichloroacetic acid
