# Corrigendum: Tengdan Capsule Prevents Hypertensive Kidney Damage in SHR by Inhibiting Periostin-Mediated Renal Fibrosis

**DOI:** 10.3389/fphar.2021.814501

**Published:** 2022-01-31

**Authors:** Xiaoli Du, Qianqian Tao, Hongxia Du, Zhenbang Zhao, Yu Dong, Shuang He, Rui Shao, Yule Wang, Wenrun Han, Xintong Wang, Yan Zhu

**Affiliations:** ^1^ Institute of Chinese Medicine, Tianjin University of Traditional Chinese Medicine, Tianjin, China; ^2^ Department of Pharmacy, Inner Mongolia Medical College, Hohhot, China; ^3^ Tianjin International Joint Academy of Biomedicine, Tianjin, China

**Keywords:** hypertension-induced kidney damage, periostin, TGFÎ^2^/SMAD signaling pathway, inflammatory response, human kidney HEK293 cells

In the original article, there was a mistake in Figure 2 as published. The **Figure 1** was accidentally duplicated in the proof stage. The correct Figure 2 appears below.

**FIGURE 2:**
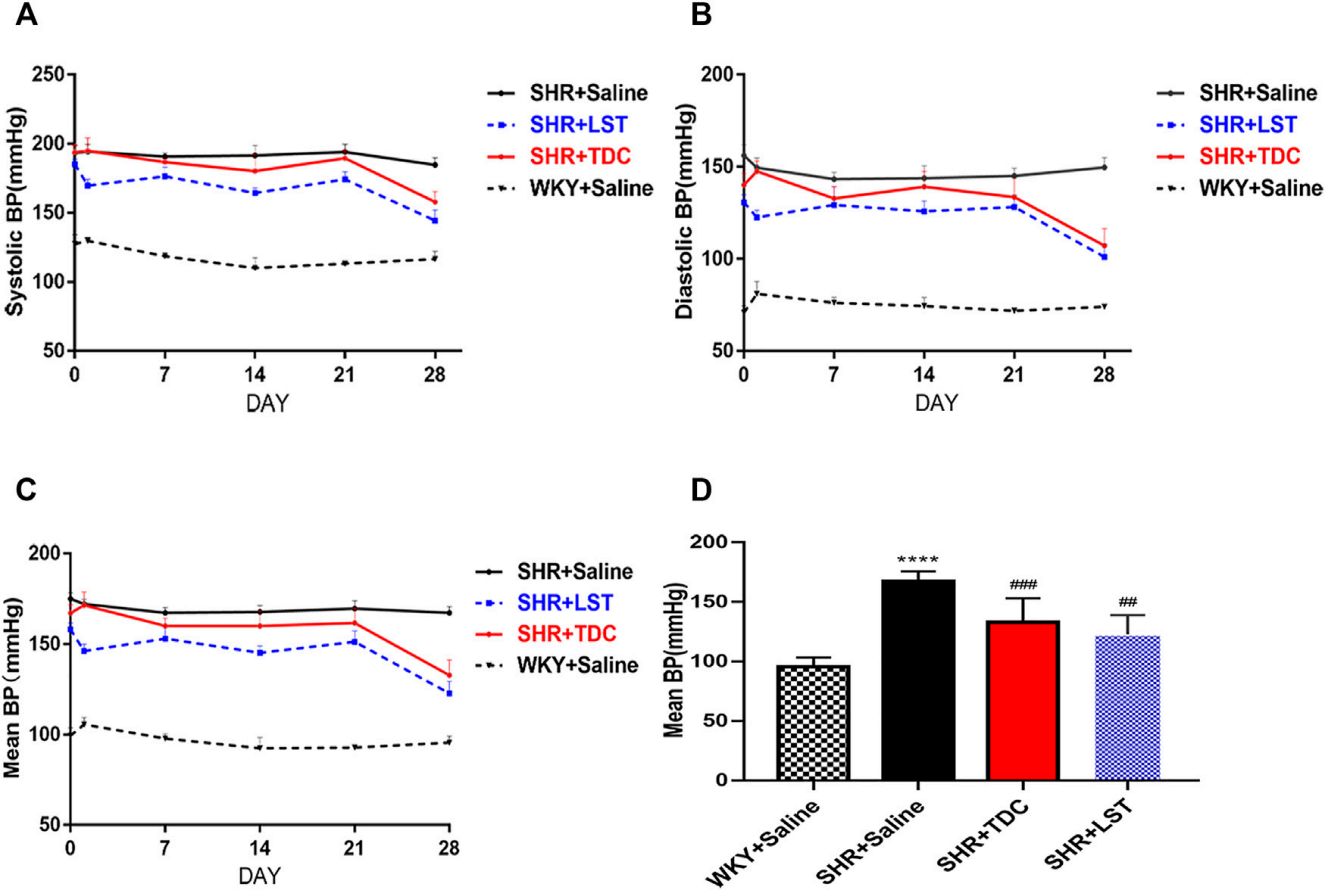
Effects of TDC on noninvasive blood pressure in SHR **(A–C)** Representative data plots of SBP, DBP, and MBP measurements, respectively, over 4 weeks using the noninvasive tail-cuff method in conscious rats **(D)** Bar graph comparison of the MBP in the four rat groups at day 28. Data are expressed as mean ± SD, n = 5–6. ****p < 0.0001 vs. WKY rats; ##p < 0.01, ###p < 0.001 vs. SHR.>

The authors apologize for this error and state that this does not change the scientific conclusions of the article in any way. The original article has been updated.

