# Supplementary material for: Tengdan Capsule Prevents Hypertensive Kidney Damage in SHR by Inhibiting Periostin-Mediated Renal Fibrosis
Source: Front Pharmacol. 2021 May 18;12:638298. doi: 10.3389/fphar.2021.638298 (PMC8167194; doi:10.3389/fphar.2021.638298)
Supplement: Supplementary file 1 [file DataSheet1.pdf]

Primer	Sequence (5'-3')
Periostin	Forward:TTATCTGCTCTGCTGCTGCTGTTC
	Reverse:ACCCCCTGATGCGGCTGTGAG
TGF-β	Forward:GACCGCAACAACGCAATCTATGAC
	Reverse:CTGGCACTGCTTCCCGAATGTC
COL1A1	Forward:TGTTGGTCCTGCTGGCAAGAATG
	Reverse:GTCACCTTGTTCGCCTGTCTCAC
GAPDH	Forward:GGCCTTCCGTGTTCCTACC
	Reverse:CGCCTGCTTCACCACCTTC

Protein IDs	Fold change (SHR+TDC/SHR)	P value	Protein symbol	Protein IDs	Fold change (SHR+TDC/SHR)	P value	Protein symbol
Q7TPI5	1.431714	0.00766525	-	A0A0G2K4U7	0.899804	0.04422991	Mtus2
Q62789	1.428659	0.00733617	Ugt2b7	Q6AY96	0.898722	0.04773918	Gtf2f1
D3ZS75	1.37997	0.0287785	Ndufc1	D3ZX79	0.898191	0.04059184	Ly6g6c
A0A0G2K906	1.338006	0.03407102	Msrb1	Q63663	0.898107	0.00382282	Gbp2
P02764	1.285768	0.01361404	Orm1	P24090	0.897675	0.04659759	**Ahsg**
A0A0G2JUH8	1.269606	0.01949442	Snx13	A0T2W6	0.89339	0.03679727	-
A0A0G2JSG8	1.269127	0.02755224	Ubd	Q4V8K5	0.892993	0.03806563	Brox
E9PTV9	1.243622	0.04663073	-	F1LQI6	0.892755	0.00587116	Zer1
P24268	1.242481	0.00231317	Ctsd	Q4V888	0.89018	0.02299819	Pip4p2
Q5XIE2	1.230079	0.03791251	Mterf2	A0A0G2K6H5	0.890054	0.04901766	Cfdp1
Q4KLZ0	1.224045	0.0009261	Vnn1	Q6LED0	0.887661	0.00023059	-
F1M4G6	1.214406	0.04434191	-	M0R3Z8	0.886748	0.02345304	Rbm15
F1LLV5	1.209815	0.0300864	Ugt2a3	Q5U318	0.885779	0.00377989	Pea15
G3V6P8	1.178551	0.04195134	**Gng12**	D3ZI99	0.885714	0.03862133	Prpf3
P22791	1.177353	0.01221345	Hmgcs2	A0A0G2K3W3	0.885459	0.003546	-
M0R789	1.175702	0.02679731	-	A0A0G2K2Q5	0.883432	3.0137E-05	Gulp1
P07896	1.164121	0.01580703	Ehhadh	Q63475	0.883371	0.04970612	Ptprn2
P16296	1.161253	0.00041667	F9	P02767	0.883005	0.0151985	Ttr
G3V734	1.160952	0.0082884	Decr1	G3V7Z4	0.880254	0.00153594	Serpine2
B1WBS5	1.156845	0.03749157	Slc5a10	O35795	0.878253	0.02482348	Entpd2
A0A0G2K3P7	1.152168	0.01700397	Atp11b	Q4KM38	0.876364	0.04588384	Srsf10
M0R3T8	1.139931	0.00593488	Ankrd26	A0A096MJZ7	0.873829	0.01874927	-
F1LRK1	1.137325	0.04505046	Atp4a	Q5I0E1	0.873767	0.04314592	**Lrg1**
P23965	1.137044	0.01617554	Eci1	Q66H74	0.872389	0.00409472	Golph3l
Q6PCU8	1.136835	0.02124075	Ndufv3	Q64240	0.866919	0.01294886	Ambp
P0CD94	1.136762	0.00315044	Uqcc3	F1M004	0.865732	0.0154494	-
A0A0G2JUA6	1.131786	0.04162833	Wbp11l1	G3V621	0.860133	0.00431382	Slc16a10
Q9R1X8	1.129143	0.04004306	PKC lambda	Q5U342	0.856582	0.01864736	Nfkbib
B2GV62	1.125038	0.00432955	Mrpl20	Q3MID6	0.856552	0.02462253	Calu
P04182	1.120533	0.03035663	Oat	B0BNG5	0.852879	0.00143169	Hif1an
D3ZKJ8	1.11849	0.02618332	Neil3	P14841	0.849448	0.02266688	**Cst3**
Q9QZK8	1.117593	0.00656768	Dnase2	A0A097BW25	0.846642	0.0174958	**Postn**
Q62669	1.112166	0.01473283	LOC103694855	Q5M8C7	0.846076	0.00182539	Txndc9
P32577	1.112109	0.03109095	Csk	D3ZZE3	0.843873	0.03677156	Armh3
D3ZX69	1.108025	0.03596447	Mrpl10	D4A333	0.843833	0.04143311	Ttc21b
P08430	1.107812	0.00150758	Ugt1a6	D3Z9I5	0.84187	0.0038116	Slc26a6
D3ZTA3	1.106939	0.04632159	Mcm3ap	A0A0G2K296	0.84145	0.00317949	Fsip2
P51647	1.105297	0.01126569	Aldh1a1	M0R8A4	0.837597	0.04931799	-
F8WG67	1.105085	0.01295011	Acot7	Q5M819	0.835226	0.00360206	Psph
Q642B6	1.103075	0.0379464	Thap4	B1WC70	0.832356	0.00058839	Ppp1r8
P11951	1.102744	0.0479872	Cox6c2	G3V8R0	0.8311	0.04558855	RGD1311703
A2VCW9	1.102701	0.0381064	Aass	A0A0G2JSM4	0.829298	0.02605195	Bet1l
A0A0G2K4G3	1.102382	0.04490562	Nedd8	A0A0G2K7L3	0.827695	0.03363186	Mpv17l
Q9EPF2	1.102056	0.00981559	Mcam	A0A0G2JTI8	0.819944	0.04272312	Enpep
Q99N97	1.101545	0.03182137	-	F1LRC6	0.819496	0.03767112	Nup188
P70552	1.100743	0.04220701	Gchfr	Q63624	0.794335	0.03171682	Scaf1
D3ZW49	1.100385	0.01287329	Tmem9b	G3V9T3	0.786093	0.04183309	Slc9a4
Q9R066	1.100329	0.01147546	Cxadr	F1M1J2	0.785658	0.04822129	Neb
				Q5J3I5	0.782481	0.04407437	Vom1r85
				Q5PQS3	0.777734	0.04472611	Veph1
				P35572	0.774587	0.00649613	Igfbp6
				P02625	0.744033	0.02680262	Pvalb
				A0A0G2JSK1	0.733933	0.00049247	Serpina3c
				Q9QX67	0.713636	0.01441138	Dap
				A0A0G2JSP8	0.711402	0.01656085	Ckm
				Q99PK0	0.372358	0.00726942	Xab2

Group	Relative collagen deposition rate [A540nm(Dose group)/A540nm(Positive medicine group)]
Control	0.85±0.00
TGF-β1	1.18±0.02
TGF-β1+SB431542	1.00±0.01
TGF-β1+LST	1.02±0.01
TGF-β1+TDCs-L	1.11±0.11
TGF-β1+TDCs-M	0.97±0.06*
TGF-β1+TDCs-H	0.93±0.03*
